# Assessment of Reliability and Quality of Videos on Medial Epicondylitis Shared on YouTube

**DOI:** 10.7759/cureus.37250

**Published:** 2023-04-07

**Authors:** Oznur Uzun

**Affiliations:** 1 Physical Medicine and Rehabilitation, Ankara City Hospital, Physical Medicine and Rehabilitation Hospital, Ministry of Health, Ankara, TUR

**Keywords:** youtube, medial epicondylitis, golfer’s elbow, global quality score, discern

## Abstract

Introduction

Video-sharing websites, especially YouTube search engine, have become popular sources for obtaining health information over the years. The reliability and quality of contents on YouTube are unpredictable and questionable. The purpose of this study was to evaluate the reliability and quality of videos on medial epicondylitis shared on the YouTube platform.

Methods

A YouTube search with the keywords “medial epicondylitis” and “golfer’s elbow” was conducted. After typing each keyword, the first 50 videos that appeared in the first three pages were evaluated. The titles and duration of the videos, the uploading sources, the time passed since the upload, number of total views, likes, dislikes and comments were recorded. All videos were analyzed and scored using the DISCERN scoring system, modified DISCERN (mod-DISCERN) scale, and the Global Quality Score (GQS).

Results

Eighty-eight videos met the inclusion criteria and were included in the study. The mean DISCERN score, mod-DISCERN score and GQS were 40.68±10.5, 1.81±0.76, and 2.72±0.9, respectively. Most of the videos had poor and very poor quality content according to the DISCERN instrument, the mod-DISCERN scale and GQS tool. The main upload sources were doctors (36.4%), physiotherapists (25%), patients (2.2%), and other (36.4%). The videos uploaded by doctors had higher quality scores than other uploading sources. Of the 88 videos, 10 were about diagnosis alone, 39 were on treatment alone, and 39 contained information about both of diagnosis and treatment. The mean DISCERN score, mod-DISCERN score and GQS of the videos on “both” were significantly higher than those “diagnosis alone” and “treatment alone” groups.

Conclusion

Nearly 80% of YouTube videos on medial epicondylitis according to the DISCERN and GQS tool, and also 97.7% of videos according to the mod-DISCERN scale had medium or poor quality. YouTube videos on medial epicondylitis could not be considered as accurate and reliable sources. Because the videos uploaded by doctors had higher quality scores, the physicians should prepare and upload more reliable and quality contents with detailed information on YouTube.

## Introduction

Epicondylitis is a prevalent disorder of the arm that affects men and women equally, predominantly between the ages of 45 and 54 years [1]. Epicondylitis is characterized by functional impairment and chronic pain in the region of the epicondyle, incited by resisted use of the flexor or extensor muscles of the wrist [1]. Medial epicondylitis, known as “golfer’s elbow,” is provoked by frequent eccentric loads on the muscles that are responsible for forearm pronation and wrist flexion. This repetitive stress leads to microtrauma of the common flexor tendon and debilitating, chronic pain at the epicondyle [2]. Despite an overall prevalence of <1%, medial epicondylitis may affect as many as 3.8% to 8.2% of patients in occupational settings. A result of common flexor tendon microtrauma and degeneration, medial epicondylitis typically occurs in the fourth through sixth decades of life, the peak working years, and equally affects men and women [3].

The Internet makes accessing quick and broad information more convenient and its use as a health information resource grows every day [4-6]. Fifty-five percent of patients indicated that they use the Internet for medical search and 60% stated that the information they found online was “same as” or “better than” the information from their doctors [7]. A recent study also showed that 80% of health seekers consult web sources to get information [8]. Video-based materials are reported to be more attractive than readable texts on the Internet [9]. Video-sharing websites, especially YouTube search engine, have become popular resources for accessing health information over the years, consequently. Because videos on YouTube are not peer-reviewed, all individuals (patients, physicians and non-physician healthcare professionals, websites and organizations) can upload contents free and easily. Thus, the reliability, quality and accuracy of the contents on YouTube are unpredictable and questionable.

YouTube, of course, may ease the understanding of their disease and may improve satisfaction in patients with medial epicondylitis in case of using right sources. On the other hand, insufficient and inaccurate contents may mislead patients and may disturb physician-patient communication. There are lots of videos about medial epicondylitis on YouTube. Accessing a reliable and good quality information in such a big data pool is hence quite important. Although several studies have examined the veracity of YouTube videos about different topics in Physical Therapy and Rehabilitation, there is no study evaluating the reliability and quality of YouTube videos on medial epicondylitis [4,10-12]. The purpose of this study was to analyze the reliability and quality of videos on medial epicondylitis shared on YouTube platform.

## Materials and methods

This study was exempt from ethical approval because of the observational design only with videos readily available to the public. A YouTube search was conducted on http://www.youtube.com with the keywords “medial epicondylitis” and “golfer’s elbow" on May 14, 2022. Before the search, the user login was ended, the search history was cleared and the filter “sort by relevance” was used to access the contents. Previous studies showed that 90% of internet users did not view more than three pages [13]. Because of that, only the first 50 videos appeared in the first three pages for each keyword were evaluated. Only videos in English were included in the study. The videos with silent content, shorter than 30 seconds, repetitive, and containing irrelevant content, turned-off comments or interactions by uploader and duplicated videos were excluded. The duration (as minutes) and the uploading sources of the videos, days on YouTube, number of views, likes, dislikes and comments were recorded. The uploading sources were classified as doctors, physiotherapists, patients, and others. The videos were categorized as “diagnosis alone”, “treatment alone”, or “both of diagnosis and treatment” according to their contents. View ratio (VR; views per day), like ratio [LR; number of likes / (number of likes + number of dislikes) × 100] and video power index (VPI) were analyzed to evaluate viewer interaction parameters. Erdem and Karaca identified VPI with the formula (LR × VR) / 100 to define the popularity of the videos [4]. The DISCERN scoring system, the modified DISCERN (mod-DISCERN) scale, and the Global Quality Score (GQS) tool were used to determine the accuracy, quality and value of the contents. DISCERN is a 3-section scoring system consisting of 16 questions to evaluate the accuracy of health information [14]. Each query in DISCERN tool was scored on a 5-point Likert scale. According to the DISCERN score, the videos were categorized as follows: excellent quality (i.e., 63-80 points), good quality (i.e., 51-62 points), medium quality (i.e., 39-50 points), poor quality (i.e., 27-38 points) or very poor quality (i.e., 16-26 points) (Table 1).

**Table 1:** DISCERN scoring system (1 point for No, 2-4 points for Partially, 5 points for Yes)

	Question			Score
Section 1	Is the publication reliable?			
1	Are the aims clear?			1-5
2	Does it achieve its aims?			1-5
3	Is it relevant?			1-5
4	Is it clear what sources of information were used to compile the publication (other than the author or producer)?			1-5
5	Is it clear when the information used or reported in the publication was produced?			1-5
6	Is it balanced and unbiased?			1-5
7	Does it provide details of additional sources of support and information?			1-5
8	Does it refer to areas of uncertainty?			1-5
Section 2	How good is the quality of information regarding treatment choices?			
9	Does it describe how each treatment works?			1-5
10	Does it describe the benefits of each treatment?			1-5
11	Does it describe the risks of each treatment?			1-5
12	Does it describe what would happen if no treatment is used?			1-5
13	Does it describe how the treatment choices affect overall quality of life?			1-5
14	Is it clear that there may be more than 1 possible treatment choice?			1-5
15	Does it provide support for shared decision-making?			1-5
Section 3	Overall rating of the publication			
16	Based on the answers to all of the above questions, rate the overall quality of the publication as a source of information about treatment choices			1-5

The mod-DISCERN scale consists of five questions adapted from the original DISCERN tool to evaluate the reliability of health information [15]. In the mod-DISCERN scale, each question was scored either 0 or 1 point. A total score of 5 indicates high reliability whereas a 0-point demonstrates low reliability in this scoring system (Table 2).

**Table 2:** The modified DISCERN tool (1 point for every Yes, 0 point for every No)

	Score	Question
	1	Are the aims clear and achieved?
	2	Are reliable sources of information used? (i.e., publication cited, speaker is board-certified rheumatologist)
	3	Is the information presented balanced and unbiased?
	4	Are additional sources of information listed for patient reference?
	5	Are areas of uncertainty mentioned?

The GQS tool consisting of five questions, was used to assess the overall quality, flow of information and educational value of the videos [16]. A 5-point GQS demonstrated the excellent quality and a 1-point GQS demonstrated low quality of the content. According to the mean GQS, videos with 4 or 5 points were categorized as high quality, those scored with 3 points were rated as medium quality, and those with 1 or 2 points were categorized as low quality (Table 3).

**Table 3:** Global Quality Score (scored based on the following characteristics)

	Score	Description
	1	Poor quality, poor flow, most information missing, not useful for patients.
	2	Generally poor, some information given but of limited use to patients.
	3	Moderate quality, some important information is adequately discussed.
	4	Good quality, good flow, most relevant information is covered, useful for patients.
	5	Excellent quality and excellent flow, very useful for patients.

Statistical analysis

Statistical analysis was performed using the Statistical Package for Social Sciences (SPSS) verson 22.0 software (IBM Corp., Armonk, NY, USA). Shapiro-Wilk test was used to determine the normality of data. Mean ± Standard Deviation (SD), median, minimum, and maximum values and frequency were given as descriptive methods. The Kendall rank correlation coefficient was performed to measure the association between the groups. The Kruskal-Wallis test was performed to determine statistically significant differences of independent variables between more than two groups. In case of requirement, Bonferroni correction was performed, and the p-value was accepted as 0.017. The results were evaluated at 95% confidence interval and a significance level of p <0.05.

## Results

Eighty-eight videos were included in the current study. The mean DISCERN score, mod-DISCERN score and GQS of all videos were 40.68±10.5, 1.81±0.76, and 2.72±0.9, respectively. The correlation between DISCERN score, mod-DISCERN score and GQS was evaluated using the Kendall rank correlation coefficient. The correlation between the mean DISCERN score and GQS was moderate to strong (p<0.001) (r=0.639). The mean mod-DISCERN score had moderate correlations with the mean DISCERN score (p<0.001) (r=0.445) and the mean GQS (p<0.001) (r=0.534). The characteristics, viewer interaction parameters, quality and reliability scores of the videos were given in Table 4.

**Table 4:** Characteristics, viewer interaction parameters, quality and reliability scores of YouTube videos

Criteria		Mean ± Standard Deviation (SD)
Time since upload (day)		1721.7 ± 344.11
Length of video (min)		7.57 ± 5.99
Number of views		150465.9 ± 37948.03
Number of likes		1560.25 ± 839.75
Number of dislikes		43.67 ± 4.98
Number of comments		88.13 ± 35.38
View ratio (VR)		13439.63 ± 9451.83
Like ratio (LR)		95.12 ± 7.43
Video Power Index (VPI)		12801.35 ± 8613.82
DISCERN score		40.68 ± 10.5
Modified-DISCERN (Mod-DISCERN) score		1.81 ± 0.76
Global Quality Score (GQS)		2.72 ± 0.9

The distribution of all videos according to the DISCERN score was as follows: 3 (3.4%) excellent, 14 (15.9%) good, 32 (36.4%) fair, 29 (32.9%) poor, and 10 (11.4%) very poor. The mean mod-DISCERN scores showed good quality in 2 (2.3%) videos, fair quality in 12 (13.6%) videos, poor quality in 41 (46.6%) videos and very poor quality in 33 (37.5%) videos. According to the mean GQS, 20 (22.7%) videos had high quality, 27 (30.7%) videos had medium quality, and 41 (46.6%) videos had low quality.

The uploading sources were doctors in 32 (36.4%), physiotherapists in 22 (25%), patients in 2 (2.2%), and others in 32 (36.4%) videos. There were no significant differences in the duration of the videos, days on YouTube, number of total views, likes, dislikes, comments, LR, VR, and VPI between the uploaders (p>0.05). The videos belonging to doctors had significantly higher DISCERN scores, mod-DISCERN scores and GQS compared to those posted by “other” (p=0.004, p=0.01, p=0.005, respectively). Doctors had significantly higher mod-DISCERN scores than physiotherapists as uploading sources (p=0.004). The videos uploaded by doctors had also insignificantly higher DISCERN scores and GQS compared to physiotherapists (p>0.05). The mean GQS and DISCERN scores of the videos uploaded by physiotherapists were insignificantly higher than those uploaded by patients (p=1.000) and others (p=1.000).

Ten (11.4%) videos included information about diagnosis alone, 39 (44.3%) were on treatment alone, and 39 (44.3%) were about both of diagnosis and treatment. The mean DISCERN score, mod-DISCERN score and GQS were similar in videos containing information about “diagnosis alone” and “treatment alone” (p=0.14, p=0.485, and p=0.447, respectively). The mean DISCERN score, mod-DISCERN score and GQS of the videos on “both of diagnosis and treatment” were significantly higher than those “diagnosis alone” (p=0.000, p=0.018 and p=0.004, respectively) or “treatment alone” groups (p=0.000, p=0.004 and p=0.000, respectively).

## Discussion

The possibility of uploading videos on YouTube for free and easily by any registered user without a standardization or peer review process results in the unpredictability and questionability of the contents. Achieving reliable and quality resources on YouTube may ease the understanding of the diagnosis and treatment in patients with medial epicondylitis. Patients with accurate and well-understood information may also have lower anxiety levels and take an active role in the treatment process [11]. However misleading and inaccurate information may worsen the relationship between the physician and the patient. Goyal et al. defined that 78% of YouTube videos on carpal tunnel syndrome have contained at least one statement that could reinforce common misconceptions and misleading information was more common in videos with lower mod-DISCERN scores [17].

There are several studies evaluating the quality of YouTube videos as information sources in different diseases [4,10,11,15]. As far as we are aware, this study is the first one in the literature evaluating the reliability and dependability of YouTube videos on medial epicondylitis. In the current study, the overall quality of videos on medial epicondylitis was poor according to the mean mod-DISCERN score and GQS, although the mean DISCERN category of all videos was fair. Additionally, nearly 80% of YouTube videos on medial epicondylitis had medium or poor quality according to the mean DISCERN score and GQS. The quality and reliability of these videos were worse according to the mod-DISCERN tool because 97.7% of videos were of moderate to very poor quality. These results suggest that health seekers on YouTube receive insufficient and unconfirmed information about medial epicondylitis.

Previous studies revealed that the uploading sources were the indicators of the content’s quality and physician-based videos provided higher-quality information [10,11]. Correspondingly, the videos uploaded by doctors had higher DISCERN scores, mod-DISCERN scores and GQS compared to physiotherapists, patients and others, in our study. Physiotherapists are the second uploading sources with high-quality information following doctors. The overall reliability and quality of YouTube videos on medial epicondylitis were even moderate to poor, although one-third of the videos were uploaded by doctors. Similarly, Celik et al. reported that YouTube videos on rotator cuff repair did not contain sufficient quality information, although the main uploading sources were physicians with higher quality scores [8]. The lower quality may be due to the tendency of physicians to record videos without medical terminology to ensure a better understanding of their contents and to increase viewer interaction parameters.

In the current study, 44.3% of the videos were on “treatment alone” and 44.3% were on “both of diagnosis and treatment”. The videos containing information about “both of diagnosis and treatment” had higher quality scores than videos on “diagnosis alone” and “treatment alone”. Although the total percentage of the videos containing information about treatment (alone or together with the diagnosis) was high, the poor quality of the contents can make it difficult for patients to achieve sufficient information. Additionally, Diaz et al. reported that 59% of online health seekers did not share what they obtained from the Internet with their doctors [7]. Considering the high viewer interaction parameters in our study, the misleading or inaccurate information can be predicted to spread faster and negatively affect the patients’ decisions on the treatment, consequently. Doctors should be aware that YouTube videos on medial epicondylitis are of poor or moderate quality. Assisting patients and offering suggestions about online health seeking may prevent patients to receive inaccurate information.

Limitations

The current study has also few limitations. First, all videos were evaluated and scored by one reviewer, but objective tools validated by several studies were used to minimize the subjectivity of the assessment. Another limitation was that only the first 50 videos for each keyword were evaluated in this study. Although this number represents a small proportion of YouTube library, previous studies showed that most of the internet users selected a search result within the first three pages [13]. Finally, a single time point was used to evaluate the quality and reliability of the videos, as the objective of the current study was to simulate an Internet user visiting YouTube for informational purposes and to analyze the results from a reviewer’s perspective. The Internet and YouTube are open-access evolving platforms with new content being uploaded every second, and the information available on YouTube is constantly changing. It is impossible to assess the quality and reliability of all YouTube videos on medial epicondylitis. Consequently, different results may be obtained in other studies conducted at different times by using the same methods.

## Conclusions

YouTube videos on medial epicondylitis could not be considered as accurate sources and health-seeking on YouTube may mislead patients with medial epicondylitis according to our study. Because the videos uploaded by doctors had higher quality scores, the physicians should prepare and upload more reliable and quality videos with detailed information on YouTube more often to educate patients about both the diagnosis and the treatment of medial epicondylitis.
